# Supplementary material for: P-TRAP: a Panicle Trait Phenotyping tool
Source: BMC Plant Biol. 2013 Aug 29;13:122. doi: 10.1186/1471-2229-13-122 (PMC3848748; doi:10.1186/1471-2229-13-122)
Supplement: Additional file 7 — P-TRAP source code. The Netbeans project that contains the source code of the application. [file 1471-2229-13-122-S7.zip › javadoc.zip!/javadoc/org-bioalg-imageprocessor/overview-tree.html]

Class Hierarchy (Image Processor)


JavaScript is disabled on your browser.


- Package
- Class
- Tree
- Deprecated
- Index
- Help

*org.bioalg.imageprocessor 1.0*

- Prev
- Next

- Frames
- No Frames

- All Classes

# Hierarchy For All Packages

Package Hierarchies:

- org.bioalg.imageprocessor

## Class Hierarchy

- java.lang.Object
  - org.bioalg.imageprocessor.AImage
  - org.bioalg.imageprocessor.ColoredImage
  - org.bioalg.imageprocessor.EDistanceMap
  - org.bioalg.imageprocessor.HSBPixel
  - org.bioalg.imageprocessor.ImageIO
  - org.bioalg.imageprocessor.Kernels
  - org.bioalg.imageprocessor.MathMorphology
  - org.bioalg.imageprocessor.RGBPixel

## Enum Hierarchy

- java.lang.Object
  - java.lang.Enum<E> (implements java.lang.Comparable<T>, java.io.Serializable)
    - org.bioalg.imageprocessor.MathMorphology.MorphingType
    - org.bioalg.imageprocessor.AImage.CONNECTIVITY

- Package
- Class
- Tree
- Deprecated
- Index
- Help

*org.bioalg.imageprocessor 1.0*

- Prev
- Next

- Frames
- No Frames

- All Classes
